# Spontaneous Isolated Thrombosed True Popliteal Aneurysm in an Eight-Year-Old Child: A Rare Case Report With Literature Review

**DOI:** 10.7759/cureus.28248

**Published:** 2022-08-21

**Authors:** Muhammad Nasir, Ilyas Sadiq, Chaudhary Abdul Fatir, Haseeb Mehmood Qadri

**Affiliations:** 1 General Surgery, Unit II, Lahore General Hospital, Lahore, PAK; 2 Vascular Surgery, Doctors Hospital & Medical Center, Lahore, PAK

**Keywords:** pediatric, open surgical repair, true aneurysm, spontaneous, popliteal artery aneurysm

## Abstract

Spontaneous aneurysms are rare in the pediatric age group. Aneurysms of peripheral arteries are even rarer. The diagnosis should not be missed to prevent distal limb ischemia and life-threatening complications. Hence, timely surgery to save the affected limb is advised. There is an increasing number of reported cases of such aneurysms in the English scientific literature. We present a rare case of pediatric idiopathic popliteal artery aneurysm (PAA), with no known risk factors. This scientific writing is unique in its way of reporting an idiopathic aneurysm with spontaneous onset. However, we have successfully investigated and managed the patient considering the established guidelines on aneurysmal surgery.

## Introduction

An aneurysm is defined as an irreversible, focal dilatation in the arterial wall, with the largest diameter of more than 50% of the normal. The normal diameter of the popliteal artery varies from 0.7 to 1.1 cm in a living human being. The mean age at presentation of popliteal aneurysms is 65 years, with males having a 20 times greater rate of incidence as compared to females [1]. Aneurysms of the popliteal artery (PAAs) account for 85% of all peripheral aneurysms [2]. In about 40%-50% of the cases, popliteal artery aneurysms are associated with abdominal aortic aneurysms. In half of the cases, they are bilateral. Although they account for 70% of all peripheral aneurysms, only 1% of the world population suffers from this surgical pathology. They can be saccular or fusiform in shape [3].

Various risk factors are attributable to PAAs, which include modifiable risk factors such as smoking, atherosclerosis, and connective tissue disorders including Marfan syndrome and Ehlers-Danlos syndrome. Advanced age, male gender, white race, and a family history of an aneurysmal disease are non-modifiable risk factors [4]. The etiology of popliteal artery aneurysms, and aneurysms in general, is unknown. A combination of genetic abnormalities and inflammatory processes, according to molecular research, may be the reason. Atherosclerosis and mural inflammation seem to cause decreased tensile strength, leading to the formation of an aneurysm. Atherosclerosis also increases flow turbulence distal to the site of narrowing, leading to a pathological dilatation of the arterial wall [5].

The clinical signs and symptoms of PAAs include pain or discomfort behind the knee, localized swelling behind the knee, knee pain, leg pain, and a sense of pulsation behind the knee [3]. Complications of peripheral arterial aneurysms include thrombosis and rupture, with thrombosis being more common than rupture, which is exceptionally rare [6]. Duplex ultrasonography (US) is the best screening and diagnostic imaging modality for detecting and estimating the diameter of a popliteal artery aneurysm. Other alternatives to duplex ultrasonography include computed tomography (CT) or magnetic resonance (MR) angiography, which can determine precise luminal diameter and aid in the planning of operative repair [7,8].

The management of PAAs includes conservative measures and operative intervention. All asymptomatic and symptomatic cases with a diameter of more than 2 cm require operative intervention to reduce the risk of limb ischemia and related complications [9]. Various operative interventions include an open surgical approach, endovascular approach, and intra-arterial thrombolytic therapy. The gold standard treatment for popliteal artery aneurysms is the open surgical approach [10,11].

This case report highlights the fact that pediatric popliteal aneurysms can present without a history of trauma or established risk factors, and hence, clinicians need to consider spontaneous PAAs when making the differential diagnoses of popliteal fossa swellings in children. There are insufficient data available on pediatric PAAs, and we found only four cases of idiopathic, spontaneous, thrombosed, and true popliteal artery aneurysms in the pediatric population in the English scientific literature.

## Case presentation

An eight-year-old male presented in November 2021 at Doctors Hospital & Medical Center, Lahore, with an active complaint of dull pain behind the left knee. He had a history of swelling behind the left knee for the past three months. The patient and his parents denied any history of trauma to his knee. On examination, the swelling was located in the upper part of the left popliteal fossa, 4 × 3 cm in size, spherical in shape, soft in consistency, having an expansile pulsation, fluctuant, compressible, and non-illuminant on the translucency test. It was not adherent to the overlying skin. There was no wound, sinus, or any kind of discoloration of the overlying skin. The left-sided knee was fixed in a semi-flexed posture during the entire examination. The left calf and foot were cold to touch. Left-sided dorsalis pedis and posterior tibial pulses were absent. The power of the left ankle dorsiflexor and plantar flexor muscles was 3/5. The contralateral limb was normal in all these aspects, with intact neurovascular status.

**Figure 1:**
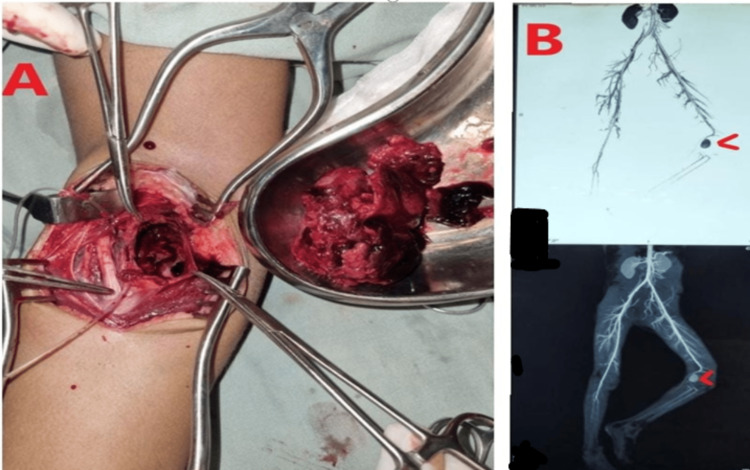
A: Aneurysm of the popliteal artery in situ. B: Isolated aneurysm on CT scan with and without contrast. CT: computed tomography

The differential diagnoses of popliteal swelling in the pediatric population under consideration were popliteal artery aneurysm, popliteal vein varix, ganglion cyst, para-meniscal cyst, and hematoma. The left knee X-ray (AP and lateral views) showed normal anatomy. However, the expansile pulsation strongly suggested a swelling of vascular origin; therefore, CT angiography was ordered. It revealed an isolated, thrombosed left popliteal artery aneurysm at the superior angle of the popliteal fossa, as shown in Figure 1. The child was provided adequate analgesia. After detailed counseling of the parents and informed consent, elective surgery was carried out in which the patient underwent a left-sided popliteal artery aneurysm repair and reverse venous grafting using the great saphenous vein from the contralateral limb. The procedure was carried out meticulously without any significant intra-operative injury to the adjacent neurovascular structures. The patient had a smooth recovery from general anesthesia and was admitted to the in-patient facility. His distal lower limb pulses recovered with a normal sensorimotor status immediately. The patient was discharged, after ensuring good postoperative wound healing and confirming normal neurological and vascular examination. We followed the patient on monthly basis for six months. His systematic clinical examinations were normal on all visits. A Doppler ultrasound was also ordered on the first postoperative visit, which was also assuring. The child is living a normal and healthy life, with a normal gait and without any postoperative neurological or vascular morbidities.

(Note: The decisions of various investigations were made considering their availability in the local healthcare facility and affordability by the parents of the patient.)

## Discussion

This case report presents a unique case of idiopathic, isolated, thrombosed, spontaneous, true aneurysm of the popliteal artery in a healthy eight-year-old male. The various risk factors for nontraumatic, spontaneous popliteal artery aneurysm, except for the male gender, such as smoking, connective tissue disorders, family history of aneurysm, and advanced age, were absent in our case [3]. On meticulous examination, the findings of swelling, pain, and cold limb, as documented in other studies, were present in our case too [4]. It is essential to focus on that idiopathic spontaneous popliteal artery aneurysms are extremely rare in the pediatric population, as in our case [4]. Therefore, the initial workup should include modalities to rule out anatomical abnormalities. CT angiography should be done to rule out other arterial aneurysms in the body, and in this case, CT angiography showed isolated, thrombosed left popliteal aneurysm only [12]. Other modalities including duplex US and MR angiography are also extremely sensitive to picking up the defect in the arterial system [4]. There are different approaches to the treatment plans of the popliteal artery aneurysm depending upon the presentation of the case and distal blood flow in the ipsilateral leg. The primary goal of the treatment is to prevent thromboembolism and amputation. The secondary goal is the prevention of aneurysmal sac enlargement [7]. There are two approaches to treating aneurysms: open surgery and endovascular repair [8]. Asymptomatic cases and aneurysms with less than 2 cm dilatation need conservative treatment only, and in all cases with symptoms and aneurysmal diameter greater than 2 cm, surgical intervention is considered the gold standard [7]. Immediate surgical exploration with excision of the diseased part and autologous reverse venous transplant (usually great saphenous vein) at its place is the warranted treatment for acute limb ischemia [7]. Intra-arterial thrombolytic therapy is not a suitable option for the pediatric age group and neither is it for the adult age group. As in our case, the patient underwent an open left-sided popliteal artery aneurysm repair and reverse venous grafting using the great saphenous vein from the contralateral limb. Patients treated electively have better primary patency with open repair than with endovascular repair [13]. Cervin et al. also demonstrated the inferiority of endovascular repair to open surgery [14]. Hence, open repair proved to be the option of choice with a good prognosis in our patient too.

## Conclusions

Thrombosed, spontaneous aneurysms should be considered a differential diagnosis in all cases of acute peripheral swellings, with no known risk factors. The open method of PAA resection with a venous graft interposition is the method of choice in cases of acute limb ischemia to save the limb. We emphasize the practice of detailed clinical examination and the opted surgical technique, as, in this case, the adequate clinical examination helped in deciding the preferred imaging modality. There are insufficient guidelines regarding the management of pediatric aneurysms. However, their treatment in tertiary care setups is highly advisable.
